# Establishment of Kawasaki disease database based on metadata standard

**DOI:** 10.1093/database/baw109

**Published:** 2016-07-26

**Authors:** Yu Rang Park, Jae-Jung Kim, Young Jo Yoon, Young-Kwang Yoon, Ha Yeong Koo, Young Mi Hong, Gi Young Jang, Soo-Yong Shin, Jong-Keuk Lee, on behalf of the Korean Kawasaki Disease Genetics Consortium

**Affiliations:** ^1^Clinical Research Center, Asan Institute of Life Sciences, Asan Medical Center, Seoul, Korea; ^2^Department of Biomedical Informatics, Asan Medical Center, Seoul, Korea; ^3^Asan Institute of Life Sciences, Asan Medical Center, Seoul, Korea; ^4^Department of Pediatrics, Ewha Womans University Hospital, Seoul, Korea; ^5^Department of Pediatrics, Korea University Hospital, Seoul, Korea

## Abstract

Kawasaki disease (KD) is a rare disease that occurs predominantly in infants and young children. To identify KD susceptibility genes and to develop a diagnostic test, a specific therapy, or prevention method, collecting KD patients’ clinical and genomic data is one of the major issues. For this purpose, Kawasaki Disease Database (KDD) was developed based on the efforts of Korean Kawasaki Disease Genetics Consortium (KKDGC). KDD is a collection of 1292 clinical data and genomic samples of 1283 patients from 13 KKDGC-participating hospitals. Each sample contains the relevant clinical data, genomic DNA and plasma samples isolated from patients’ blood, omics data and KD-associated genotype data. Clinical data was collected and saved using the common data elements based on the ISO/IEC 11179 metadata standard. Two genome-wide association study data of total 482 samples and whole exome sequencing data of 12 samples were also collected. In addition, KDD includes the rare cases of KD (16 cases with family history, 46 cases with recurrence, 119 cases with intravenous immunoglobulin non-responsiveness, and 52 cases with coronary artery aneurysm). As the first public database for KD, KDD can significantly facilitate KD studies. All data in KDD can be searchable and downloadable. KDD was implemented in PHP, MySQL and Apache, with all major browsers supported.

**Database URL:** http://www.kawasakidisease.kr

## Background

Kawasaki disease (KD) is an acute, self-limited vasculitis that occurs predominantly in infants and young children. It is characterized by prolonged fever; bilateral conjunctival injection; erythema of the oral mucosa, lips, and tongue; polymorphous rash; erythema of the palms and soles; and cervical lymphadenopathy (1). Frequently, KD is complicated by coronary artery lesions (CALs), with coronary artery aneurysms (CAAs) developing in approximately 15–25% of untreated children (2), making this disease the leading cause of acquired heart disease among children in developed countries. Although clinical and epidemiological features suggest an infectious trigger, no pathogen has been isolated to date. Because the etiology of KD is largely unknown, no diagnostic test is available (3). Multiple lines of evidence suggest that genetic determinants contribute to KD susceptibility and outcome. For example, Asian countries have a much higher incidence of KD than Western countries, and Asian-Americans also have a similar incidence to Asians (4–7). In particular, Korea has the second highest annual incidence rate after Japan (8). Although KD occurs more frequently in Asian countries, it is a relatively rare disease, with an incidence in Korea of 134.4 per 100 000 children under 5 years old (8). Given these low incidence rates, collecting patient samples is a major limitation for studying KD. To overcome this limitation, Korean Kawasaki Disease Genetics Consortium (KKDGC) was formed in 2008 to collect KD patients’ clinical data and genomic samples for large-scale genetic studies.

To facilitate the usage of the clinical and genetic data collected by KKDGC, Kawasaki Disease Database (KDD) was developed. When developing KDD, we decided to adopt metadata standard, ISO/IEC 11179, for easy and accurate data management. ISO/IEC 11179 specifies a metadata model for representing the common data elements (CDEs) that are logical data units to provide the definitions of data including an identifier and response option values for indicating the value type; and detailed information for representing data concepts and its semantics (9). The numerous large scale of clinical studies has developed standardized data elements using ISO/IEC 11179 by researchers and domain exports for providing unified data collection, and facilitating data sharing, for example, the Cancer Bioinformatics Grid (10, 11), the National Institute of Neurological Disorders and Stroke (NINDS) common data element project (12–15), the Parkinson Disease Biomarker Program (PDBP) (16–18) and as well as a number of other clinical CDE for a variety of different purposes (16–18). Following these trend, we constructed a set of KD CDE as a common language across KD re**s**earch in compliance with ISO/IEC 11179 basic attributes. KDD was developed by obtaining clinical data, sample resources, genomic data and specific genotypes of interest. To promote KD research, KDD is open to public and all contents are freely downloadable. In this paper, a detailed description of KDD with their CDEs will be introduced.

## Methods

### Development of common data elements for Kawasaki disease

KKDGC was formed in May 2008 and continued up until the present with 13 tertiary hospitals in Korea. The members of KKDGC built a data collection form by collecting clinical data of KD patients using 51 variables (19). It is composed by the following categories: (i) personal information, (ii) clinical signs and symptoms, (iii) echocardiogram finding, (iv) treatment, (v) family history and recurrence and (vi) laboratory test data. Several discussions and meeting were held for building a data collection form that represents genotype and omics data. Based on this data collection form (available as Supplementary Material), a set of CDEs were developed to analyze KD clinical and genomic data from multiple hospitals. The CDEs were built by two medical informatics professionals who independently reviewed and extracted data elements in compliance with metadata standard (ISO/IEC 11179 basic attributes). For comparative research across different organizations in KD, we selected ‘Core’ CDEs which were relevant to most of the study, such as patient demographic, sample, laboratory and genotype data. The terminology from NCI thesaurus version 15.12d is used for representing semantic of CDE such as the name of CDE, concept, and classification scheme. The NCI CDE and thesaurus is a de facto contents standard in biomedical research by providing a quantity of CDEs for various clinical domains. The NCI CDE has 51 461 CDEs for capturing data from biomedical research in NCI. The defined KD CDEs were mapped to National Cancer Institution (NCI) CDEs. Any discrepancies were resolved through discussion and consensus between two medical informatics professionals.

### Collection of data

Children with KD according to the diagnostic criteria of the American Heart Association (20) have been recruited from 13 KKDGC-participating hospitals. Currently, a total of 1292 cases from 1,283 patients have been collected. Particularly, in case of recurrence, patient’s clinical data and blood samples were collected twice. Each case includes patients’ clinical data and approximately 5 ml of blood samples for genomic DNA and plasma isolation. All clinical data was anonymized to protect patient privacy. Two genome-wide association studies (GWAS) and whole exome sequencing (WES) were performed by KKDGC. These three genomic experiments are defined as omics data in our KDD. One GWAS used Affymetrix SNP array 6.0 in 186 KD patients and 600 healthy controls (21). The other GWAS used Illumina HumanOmni1-Quad BeadChip in 296 KD cases and 1000 healthy controls. These KD cases used for GWAS include 16 cases with family history, 46 cases with recurrence, 119 cases with intravenous immunoglobulin (IVIG) non-responsiveness, and 52 cases with CAAs (diameter > 5 mm). In addition, one pilot WES data was generated on genomic DNA samples obtained from 12 KD patients using Agilent SureSelect Human All Exon 50M kit and Illumina HiSeq 2000 platform.

### Implementation

**Figure 1.**
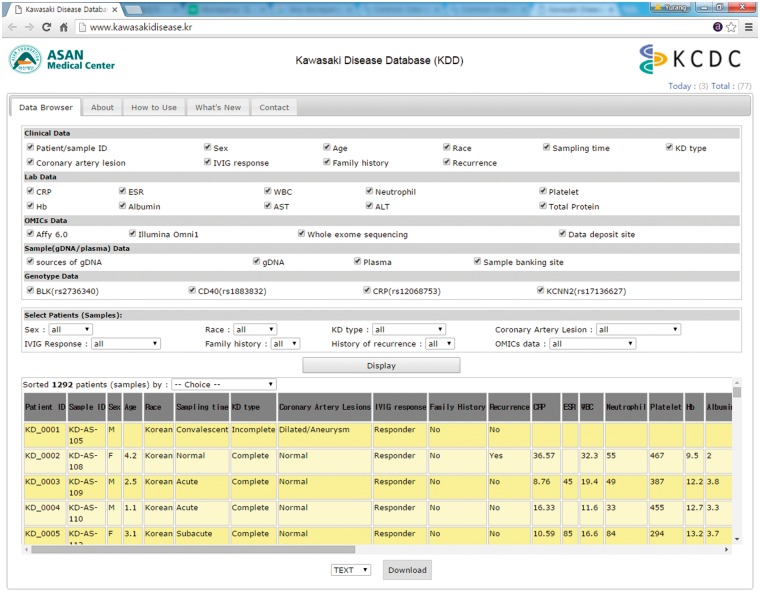
Screenshot of KDD Data Browser.

The KDD was built in Linux Cent OS 5.2 server using PHP 5.3.3, MySQL 5.1.73, and Apache 5.5. KDD can be accessed via all major browsers such as Chrome, Safari, Internet Explorer, Edge and Firefox. Clinical data, genomic data, sample data and genotype data can be selected or deselected by checking the column on the KDD web site as in Figure 1. In addition, clinical data and sample data can be sorted out by filtering options with multiple variables, such as sex, race, KD type, coronary artery lesions, IVIG response, family history, and history of recurrence and/or omics and genotyping data. All data from KDD can be freely downloaded as a CSV format via email. There is no restriction on the use of the data available from KDD.

### Privacy protection

To protect the privacy of patients who participated in our project, initially all experiments and protocols were approved by the Institutional Review Board of each institute which participated to collect clinical and blood samples. In addition, the development of KDD was approved by Institutional Review Board of Asan Medical Center (2014-0823). When we developed KDD, we excluded all 18 HIPAA protected health information such as patient’s name, date of birth, and date of admission.

## Results

### Common data elements for Kawasaki disease

A total of 63 CDEs have been defined for sharing clinical and genomic data of KD collected in 13 KKDGC-participating hospitals. The detailed information about all KD CDEs is shown in Supplementary Table 1. Among 63 CDEs, we selected 33 core CDEs to construct KDD as an open-access database. The remaining 30 CDEs and related data are internally kept for privacy and other reasons. The 33 core CDEs are shown in Supplementary Table 2. The core CDEs are categorized by the following seven classification schemes: patient demographic information, sample data, echocardiogram finding, treatment, laboratory data, omics data and genotype data. Each data element has a detailed description, semantics and syntactic information. The value domain represents syntactic information of data element whether it is enumerated or non-enumerated such as text entities or numeric values. The semantics of data element represented in object and property class used controlled vocabulary from the NCI thesaurus. All data element is linked to its relevant classification scheme. We found that, among the 33 KD CDEs, 16 matched with NCI CDEs as in Supplementary Table 2.

### Kawasaki disease database

KDD consists of the following five parts: (i) clinical data, (ii) lab data, (iii) omics (genomic) data, (iv) sample (gDNA/plasma) data, and (v) genetic variant (genotype) data. Table 1 shows the demographic and clinical characteristics of patients in this database. Of the 1292 cases, 785 are males and 507 are females. Their mean age is 2.94 years. Of these, 344 (26.7%) had incomplete KD characterized by fever lasting 5 days or longer and two or three of the five principal clinical features of KD. A family history of KD was reported for 18 patients (1.4%), and 58 patients (4.5%) had recurrent KD. As expected, we observed strong inflammatory reactions in the KD patients such as high C-reactive protein (CRP) concentration, erythrocyte sedimentation rate (ESR), and white blood cell (WBC) count, with some increase in the mean platelet count as well as the aspartate aminotransferase (AST) and alanine aminotransferase (ALT) concentrations. Clinical data are captured by above 51 CDEs for KD. Among 1283 KD patients’ genomic DNA samples, a total of 449 of the KD patients have genomic data such as Affymetrix SNP array 6.0 data (*n* = 185), Illumina HumanOmni1-Quad BeadChip data (*n* = 296), or whole exome sequencing data (*n* = 12). All genomic raw data and blood samples (genomic DNA and plasma) were deposited at either KKDGC and/or Korea BioBank Network (KBN; https://koreabiobank.re.kr/). The deposited data can be obtained for research by contacting KKDGC or KBN. The genotype data of significantly KD associated variants, including *BLK* (rs2736340) (22), *CD40* (rs1883832), *CRP* (rs12068753) (23), and *KCNN2* (rs17136627) (24) is also in this database. The detailed information on specific genetic variants can be found in our previous publications (22–24).

**Table 1.** Demographic and clinical characteristics of the patients in KDD

	KD^a^ (*n* = 1292)
Age (years)	2.94 ± 2.18
Male: *n* (%)	785 (60.8%)
Incomplete KD: *n* (%)	344 (26.7%)
CAL: *n* (%)	275 (21.5%)
IVIG non-responders: *n* (%)	154 (12.4%)
Family history: *n* (%)	18 (1.4%)
Recurrence: *n* (%)	58 (4.5%)
Baseline laboratory findings	
CRP (mg/dl)	8.08 ± 6.46
ESR (mm/h)	54.7 ± 31.7
WBC (×10^9^/l)	14.1 ± 6.58
Neutrophil (%)	63.2 ± 16.9
Platelet (×10^9^/l)	341.3 ± 111.3
Hb (g/dl)	11.4 ± 1.00
AST (IU/l)	74.1 ± 124.5
ALT (IU/l)	86.3 ± 132.6
Albumin (g/dl)	3.84 ± 0.50
Total protein (g/dl)	6.64 ± 0.69

Abbreviations: AST, aspartate aminotransferase; ALT, alanine aminotransferase; CAL, coronary artery lesion; CRP, C-reactive protein; ESR, erythrocyte sedimentation rate; KD, Kawasaki disease; Hb, hemoglobin; IVIG, intravenous immunoglobulin; WBC, white blood cell; .

^a^Quantitative variables are shown as means ± standard deviation.

To demonstrate the value of KDD, a web-survey was conducted for KD researchers and 16 researchers rated the usefulness and merits of KDD on a scale from 1 (bad) to 5 (good). Table 2 shows the results of survey. Researchers rated overall impression of KDD as 4.6. They also rated design, easiness, and contents of KDD as 4.5, 4.3 and 4.3, respectively. The lowest rate is 4.1 which is ‘It was straightforward to use KDD’. The survey implies researchers quite satisfied with KDD.

**Table 2.** The results of KDD usability survey

Category	Questions	Rate (1–5)^a^
Overall impression	Overall, I am satisfied with Kawasaki Disease Database (KDD)	4.6
	I would recommend KDD to other KD researchers	4.6
Design of KDD	It was easy to find and read the necessary data	4.4
	Data was well organized	4.8
	Data search and filtering was easy and useful	4.3
	Data download process was easy and useful	4.3
Easiness of KDD	It was easy to learn how to use KDD	4.5
	It was straightforward to use KDD	4.1
Contents of KDD	Clinical data	4.2
	Lab data	4.4
	OMICS data	4.4
	Sample (gDNA/plasma) data	4.3
	Genotype data	4.3

^a^Average from 16 researchers.

## Conclusions

KDD is the first public database for KD researchers. Lots of research articles have been published to find a potential disease cause, a treatment, and a prevention method. However, the well-organized and freely-accessible KD database was not developed except for KDD.

To facilitate KD studies, KDD has several important features. First, this database contains 1,283 patients’ KD data collected for >8 years from 13 different hospitals in Korea since 2008. It also contains information on rare cases: 16 cases with family history, 46 cases with recurrence, 119 cases with IVIG non-responsiveness, and 52 cases with CAAs (diameter > 5 mm). Second, KDD categorized all clinical data, genomic sample resources, genomic data, and specific genotypes of interests for easy search and access. Third, this database is constructed based on metadata standard, ISO/IEC 11179. The use of standardized CDEs which are mapped to NCI CDEs can provide a number of benefits for investigators and the research community. Fourth, all KD data can be easily sorted out by several filtering options to know the current status of specific subgroup of KD patient samples. Last, all de-identified data in KDD can be downloadable without restriction.

We expect that KDD will be utilized as a very useful resource portal site for KD research. With KDD, both clinical and genetic studies can be performed. For example, the researchers can investigate the ethnic difference in clinical outcomes of KD and develop prediction model for clinical outcomes based on specific clinical risk factors selected from KDD. Particularly, several different risk scores for predicting IVIG resistance have been proposed (25–30), mainly based on clinical and lab data, with limited usability due to ethnic group differences and low reproducibility under very heterogeneous clinical conditions (31–32). In this case, our clinical data and lab data can be used to investigate and validate the risk score prediction model for cross-ethnic group validation and another independent large-scale replication studies. Furthermore, in the future, they can also investigate gene-gene and/or gene-environment (clinical subgroup) interactions using genetic data and clinical data of KDD.

Survey demonstrates the KD researchers quite satisfied with KDD. However, there are still limitations on KDD. The current version of KDD only includes data from a single country. To overcome this limitation, we will try and hope to collect more KD data from other countries. More KD data deposit from other countries into KDD will make this database much useful for international KD studies. In addition, limited genomic and genotype data are available. We plan to add more WES data and genotype data to enrich database contents.

To improve the utility of KDD for KD research community, we initially got some feedback on this database from the researchers at Korean Kawasaki Disease Genetics Consortium by emails. To demonstrate the usability of KDD, we presented KDD at the 11th Kawasaki Disease Symposium organized by the Korean Society of Kawasaki Disease on May 28, 2016 and will introduce KDD at the international conferences such as the International Kawasaki Disease Symposium.

## Supplementary data

Supplementary data are available at *Database* Online.

Supplementary Data

## Korean Kawasaki Disease Genetics Consortium

Jeong-Jin Yoo, In-Sook Park, Soo-Jong Hong, Kwi-Joo Kim (Department of Pediatrics, Asan Medical Center, Seoul, Korea); Jong-Keuk Lee, Jae-Jung Kim, Young-Mi Park (Asan Institute for Life Sciences, University of Ulsan College of Medicine, Seoul, Korea); Young Mi Hong, Saejung Sohn (Department of Pediatrics, Ewha Womans University Hospital, Seoul, Korea); Gi Young Jang, Kee-Soo Ha, Hyo-Kyoung Nam, Jung-Hye Byeon (Department of Pediatrics, Korea University Hospital, Seoul, Korea); Sin Weon Yun (Department of Pediatrics, Chung-Ang University Hospital, Seoul, Korea); Myung Ki Han (Department of Pediatrics, University of Ulsan, Gangneung Asan Hospital, Gangneung, Korea); Kyung-Yil Lee, Jung-Woo Rhim (Department of Pediatrics, The Catholic University of Korea, St. Mary’s Hospital, Daejeon, Korea); Min Seob Song (Department of Pediatrics, Inje University Paik Hospital, Busan, Korea); Hyoung Doo Lee (Department of Pediatrics, Pusan National University Hospital, Busan, Korea); Dong Soo Kim (Department of Pediatrics, Yonsei University College of Medicine, Severance Children’s Hospital, Seoul, Korea); Kyung Lim Yoon (Department of Pediatrics, Kyung Hee University Hospital at Gangdong, Seoul, Korea); Hong-Ryang Kil (Department of Pediatrics, Chungnam National University Hospital, Daejeon, Korea); Gi Beom Kim (Department of Pediatrics, Seoul National University Children’s Hospital, Seoul, Korea);; Jae-Moo Lee, Jong-Duk Kim (Seoul Clinical Laboratories, Seoul, Korea).
