# Supplementary material for: Establishment of Kawasaki disease database based on metadata standard
Source: Database (Oxford). 2016 Jul 26;2016:baw109. doi: 10.1093/database/baw109 (PMC4962667; doi:10.1093/database/baw109)
Supplement: Supplementary Data [file supp_2016_baw109_index.html]

Supplementary Data 

# Establishment of Kawasaki disease database based on metadata standard

## Supplementary Data

files

- Supplementary Data - zip file
