# Supplementary material for: Establishment of Kawasaki disease database based on metadata standard
Source: Database (Oxford). 2016 Jul 26;2016:baw109. doi: 10.1093/database/baw109 (PMC4962667; doi:10.1093/database/baw109)
Supplement: Supplementary Data [file supp_baw109_suppl_data.zip › Supplement Material 1 - Kawasaki Disease Clinical Data Collection Sheet.doc]

**<Kawasaki Disease Clinical Data Collection Sheet>**

**Personal Information**

- Patient ID: ____________________
- Patient initial: ____________________
- Sex: (1) Male (2) Female
- Race: (1) Korean (2) Others: ____________
- Date of birth (yyyy-mm-dd): ______________________

**Hospital admission information**

- Country Name: _________________________________
- Hospital Name: ________________________________
- Date of admission (yyyy-mm-dd): _________________
- Date of discharge (yyyy-mm-dd): _________________
- Date of Sampling (yyyy-mm-dd): ___________
- KD Sample ID: ___KD-_________________
- Kawasaki Disease severity: (1) Acute (2) Subacute (3) Convalescent (4) Normal

**History of disease**

- Family history (patient sibling): (1) No (2) Yes

If yes, write relationship between patients: ________________

- History of recurrence: (1) No (2) Yes

If yes, Data of previous attack (yyyy-mm-dd): _________________

**Clinical signs & symptoms**

- Fever≥5 Days: (1) No (2) Yes
  - If Yes, date of onset: ______________ total: _____ days
- Conjunctival injection: (1) No (2) Yes
- Cervical Lymphadenopathy (>1.5 cm): (1) No (2) Yes
- Polymorphous skin rashes: (1) No (2) Yes
- Abnormalities of Lip or Oral Mucosa: (1) No (2) Yes

*(Strawberry tongue/fissured lips/injected pharynx)*

- Abnormalities of extremities: (1) No (2) Yes

*(edema of palm & soles/desquamation of finger tips)*

- Type of Kawasakidisease: (1) incomplete (fever + ≥ 2 criteria) (2) complete (fever + ≥ criteria)
- Other clinical signs or symptoms: __________________________

**Echocardiogram finding**

- Coronary artery: (1) normal (2) Dilated/Aneurysm
- Diameter (at worst): _____________________mm
- Data of Echo(yyyy-mm-dd): _________________

**Treatment**

- Standard treatment (2 g/kg IVIG): (1) No (2) Yes
- Date of IVIG infusion (yyyy-mm-dd): _________________
- Response of IVIG treatment

(1) Responder (disappearance of fever after treatment)

(2) Retreated (= Non-responder) (write the retreatment: ____________________________________)

(3)Not treated: (Reason: ___________________________________________________________)

- If patient doesn’t get standard treatment, write non-standard primary treatment:

_________________________________________________________________________________

- Date of Non-standard primary treatment (yyyy-mm-dd): __________________________

Laboratory date

- Date of measurement (yyyy-mm-dd): ____________________

| CRP: | (mg/L) | ESR: | (mm/hr) |
| --- | --- | --- | --- |
| White blood cell count: | (x10^9^/L) | Neutrophil: | (%) |
| Platelet count: | (x10^9^/L) | Hb: | (g/L) |
| Albumin: | (mg/L) | AST/ALT: | (IU/L) |
| Total protein: | (mg/L) | Other lab test: __________ Result: ___________ | |
